# Phase Separation Driven On-Demand Debondable Waterborne Pressure-Sensitive Adhesives

**DOI:** 10.3390/polym10090975

**Published:** 2018-09-02

**Authors:** Ehsan Mehravar, Michael A. Gross, Gracia Patricia Leal, Bernd Reck, Jose R. Leiza, José M. Asua

**Affiliations:** 1 POLYMAT and Kimika Aplikatua Saila, Kimika Zientzien Fakultatea, University of the Basque Country UPV/EHU, Joxe Mari Korta Zentroa, Tolosa Hiribidea 72, 20018 Donostia-San Sebastian, Spain; ehsan.mehravar@ehu.eus (E.M.); graciapatricia.leal@ehu.eus (G.P.L.); jrleiza@ehu.eus (J.R.L.); 2 BASF SE, Dispersions & Colloidal Materials GMD-B001, Carl-Bosch-Strasse 38, 67056 Ludwigshafen, Germany; michael.a.gross@basf.com (M.A.G.); bernd.reck@basf.com (B.R.)

**Keywords:** pressure-sensitive adhesives, crystallinity, core-shell, blend, phase migration, debondability

## Abstract

A waterborne pressure-sensitive adhesive (PSA) that shows high adhesive performance and easy debondability on demand without leaving residues on the substrate (adhesive failure) has been developed. A key component of the PSA is a semicrystalline phase that is beneficial for the adhesive properties and that becomes fluid when heated above the melting temperature. Migration of this liquid-like polymer to the substrate-adhesive interface and hardening upon cooling results in a hard non-tacky interface that facilitates debonding. The effect of the particle morphology on the debonding ability is discussed.

## 1. Introduction

Pressure-sensitive adhesives (PSAs) are materials that are involved in many aspects of our everyday life. As they are used in small amounts, their recycling is not viable; however, they may interfere with the recycling of other materials [1]. In this context, the possibility of switching off the adhesion on demand will be very valuable because it will simplify the recycling of goods at the end of their lifespan [2,3,4,5]. For this application, the most useful property is debondability, namely, the possibility of removing a bonded adhesive by adhesive failure. Therefore, transformation of the PSA before its use to reduce its adhesive performance, which has also been referred as switching-off [6,7], is not considered here. Several strategies for achieving adhesive debondability have been reported, and these are divided in physical and chemical methods [8]. Physical approaches play with aspects such as the backing material, substrate deformation and rate effects and they are not of general use. Chemical approaches are much more interesting and mainly consist of introducing a switching moiety in the otherwise strong adhesive. This moiety is activated by external stimulus (most often light and heat). One strategy that has been employed for solventborne adhesives is including photo-crosslinkable reagents in the adhesive microstructure [9,10,11,12]. Webster and coworkers [9,10] and Lee et al. [12] used UV light to switch-off the adhesion of acrylic-based PSAs by cross-linking of a soft polymer. The basis of their switchable adhesive was an acrylic copolymer containing pendant methacrylate groups capable of crosslinking upon exposure to light when combined with a suitable photoinitiator. Trenor et al. [11] used UV light to switch-off photocrosslinkable PSAs synthesized with coumarin functionalities. The formation of reversible coumarin crosslinks gelled the PSA and reduced peel strength from 1.62 to 0.05 N/mm. Another strategy to achieve a switchable-off PSA is using water-soluble tackifiers in PSA formulation [13]. The water-soluble tackifiers are low molecular weight materials, typically ethoxylated alkyl phenols of polyethylene glycols. PSAs containing water-soluble tackifiers are tacky in the dry state. When water contacts the adhesive, it is readily absorbed by the hydrophilic parts, and phase separation occurs between the water-soluble tackifier and the base polymer. The soluble tackifier migrates to the adhesive/substrate interface, forming a weak layer and leading to easy removal. It has been reported that the peel strength reductions of about 90% have been achieved after one minute of wetting [13]. However, the usefulness of water-sensitive PSAs for general purpose applications is limited. Another stimulus used to trigger PSAs switch-off is temperature. Sato and coworkers designed switchable-off PSAs using block copolymers containing a soft acrylic segment and a reactive (decomposable) polymer segment (poly(tert-butyl acrylate) or poly(isobornyl acrylate)) in the presence of a photoacid generator [14,15,16]. Degradable polymers (polyperoxides) were also used [17]. It has been reported that a drastic change in the adhesive strength was observed in response to UV irradiation and the subsequent heating. However, volatile organic compounds were produced. Nishiyama et al. [18] and Ishikawa et al. [19] developed adhesives containing thermally expansive particles that separate the bonded building materials when heated at 100 °C.

Semicrystalline polymers have also been used. A cool-off adhesive containing crystallizable long pendant chains has been developed for skin applications [20]. Above the melting temperature the adhesive behaves as a PSA but upon cooling the adhesive becomes hard and non-tacky. The applicability of this cool-off approach to general purpose adhesives is limited, as most of them are used at temperatures lower than the casting temperature. Agirre et al. [21] developed high-performance waterborne PSAs containing semicrystalline poly(stearyl acrylate), poly(SA). Surprisingly, it was reported that blends of poly(SA) rich latexes with latexes devoid of poly(SA) had better adhesive properties than latexes of the same overall composition formed by hybrid core-shell particles. The reason for this behavior has been unveiled recently [22]. A characteristic of these PSAs is that peel and shear resistance abruptly drop when they are heated above the melting temperature of the poly(SA) [21,22]. The reason is that once the crystalline phase is melted, the adhesive becomes liquid-like and loses cohesion. However, the usefulness of this behavior for recycling is limited, because at that temperature, the adhesives are liquid-like materials and suffered cohesive failure; namely, part of the adhesive remains on both substrates.

This work reports on waterborne PSAs that combine high adhesive performance and debondability with adhesive failure. A key component of the PSA is a semicrystalline phase that is beneficial for the adhesive properties as it provides cohesion to the adhesive and can easily be modified by heating above its melting temperature, T_m_. The debondability mechanism is based on the migration of the crystalline domains to the substrate-adhesive interface when the temperature is above T_m_. Recrystallization occurs upon cooling, and the polymer at the interface becomes hard and non-tacky allowing easy debonding.

## 2. Experimental Section

### 2.1. Latex Synthesis

In this work, blends of hard semicrystalline latexes and regular (meth)acrylate PSA latexes were used. There are two reasons for this choice. Firstly, that the adhesive performance of blends is much better than that of hybrid latexes of the same overall composition [21,22]. Secondly, that phase migration is sought, and it has been demonstrated that in films cast from waterborne latexes, phase migration is maximized when the mobile phase is not encapsulated by the harder phase and when the phases are incompatible [23]. Because of the high hydrophobicity of poly(SA), the hybrid latexes containing poly(SA) are core-shell particles with the poly(SA) in the core and the (meth)acrylate polymer in the shell. This means that upon melting, the liquid poly(SA) is surrounded by the less mobile (meth)acrylate polymer. In addition, as poly(SA) contains labile tertiary hydrogens that can be abstracted, it is prone to suffer transfer to polymer that leads to the formation of grafted polymer. This increases the compatibility between poly(SA) and the (meth)acrylate polymer reducing phase migration and separation.

The synthesized latexes and the blends are summarized in Table 1. For the sake of comparison and to demonstrate that phase migration is limited for hybrids, two core-shell latexes were prepared. Latex 1 was obtained by batch miniemulsion polymerization of stearyl acrylate (T = 70 °C, Disponil FES 32 (BASF, Ludwigshafen, Germany) (2 wt % based on SA) as surfactant, NaHCO_3_ (BASF, Ludwigshafen, Germany) (0.16 wt % based on SA) as buffer, and potassium persulfate (KPS, Fluka, Neu-Ulm, Germany) (0.5 wt % based on SA) as initiator). Latex 2 was synthesized in a 2.5 h seeded semicontinuous emulsion polymerization of 2-ethylhexyl acrylate (2EHA, BASF, Ludwigshafen, Germany), methyl methacrylate (MMA, BASF, Ludwigshafen, Germany), and methacrylic acid (MAA, BASF, Ludwigshafen, Germany) (SC(M)A: 2EHA/MMA/MAA = 84/15/1 by weight) using a poly(styrene) seed (0.1 wt % based on monomers; seed diameter = 30 nm). Latexes 7 and 8 were also obtained by seeded semicontinuous emulsion polymerization as described in ref. [22]. Therefore, the reactor was charged with poly(SA) latex (Latex 1) and then the pre-emulsion of SC(M)A and KPS solution were fed to the reactor over 3.5 h. The reactions were carried out at 85 °C using Disponil FES 32 (2 wt % based on monomer) as surfactant and KPS (0.5 wt % based on monomer) as initiator. The solids content of all the latexes was 45 wt %.

### 2.2. Adhesive Characterization

55-μm-thick adhesive films (cast and dried at room temperature for 4 h over a flame-treated polyethylene terephthalate (PET) sheet) were prepared. Two series of experiments were carried out. The first aimed at studying the effect of the poly(SA) migration to the air-adhesive film interface caused by annealing on the adhesive properties of the tape. For this, the tapes (adhesive on PET) were annealed at 60 °C for 10 min before attaching it to the stainless steel substrate. The work of adhesion and peel strength and the annealed films were determined at 23 °C and 55% relative humidity and compared with the performance of the non-annealed adhesives.

**Scheme 1:**
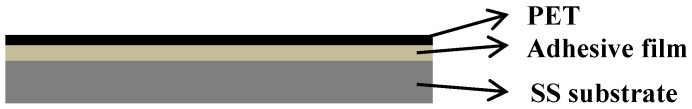
The adhered PSA tape on the stainless steel substrate.

The second series aimed at studying the effect of the debondability of the adhesives already attached to stainless steel substrates. For that the PSA tape strips (12.5 cm × 2.5 cm) on a stainless steel (SS) substrate under pressure (4 passes of a 2 kg roller) at 23 °C followed by a 20 min build up in adhesion. The PET-adhesive-SS system (Scheme 1) was annealed at 60 °C (just above the melting temperature (51 °C) of the semicrystalline phase [22,24,25] for 10 min and then allowed to cool down to room temperature. Peel strength before and after annealing was measured. SEM micrographs of the detached adhesives films were taken (Hitachi TM3030 scanning electron microscope, Hitachi, Tokyo, Japan; images were obtained at 15 kV from gold coated samples).

The work of adhesion was determined in a probe-tack measurement (Avery method [26] using a MicroSystems Texture Analyzer (Food Tech Corp, VA, USA) with a 1-in. spherical stainless steel probe. The peel adhesion was determined by means of the 180° peel test [27]. Film morphology was determined by AFM and TEM images of the cross-section of the films before and after annealing. TEM analysis was carried out with a TecnaiTM G2 20 Twin device at 200 kV (FEI Electron Microscope, FEI Comopany, Hillsboro, OR, USA) and the films were cryosectioned with a Leica EMUC6 cryoultramicrotome (Lecia Microsystems, Wetzlar, Germany) at −80 °C with a Diatome 45° diamond knife. AFM analysis was carried out with a Dimension ICON AFM with a Nanoscope V controller (Bruker, Billerica, Massachusetts, USA) operating in tapping mode. An integrated silicon tip/cantilever with a resonance frequency of around 300 kHz was used, performing measurements at a scan rate of 1 Hz/sec with 512 scan lines. The fractured surfaces were prepared by cryogenic fracturing at −80 °C.

## 3. Results and Discussion

**Figure 1:**
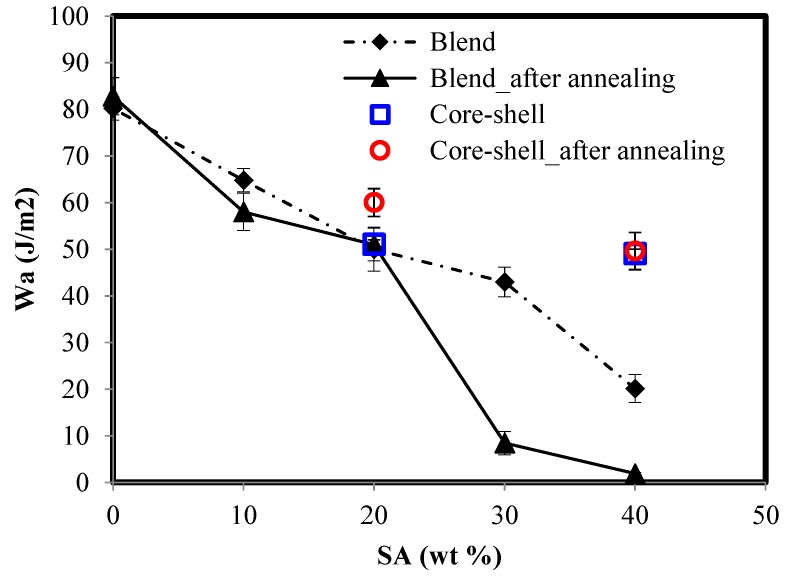
Effect of annealing on the work of adhesion of the adhesive tapes cast from blends and core-shell latexes.

**Figure 2:**
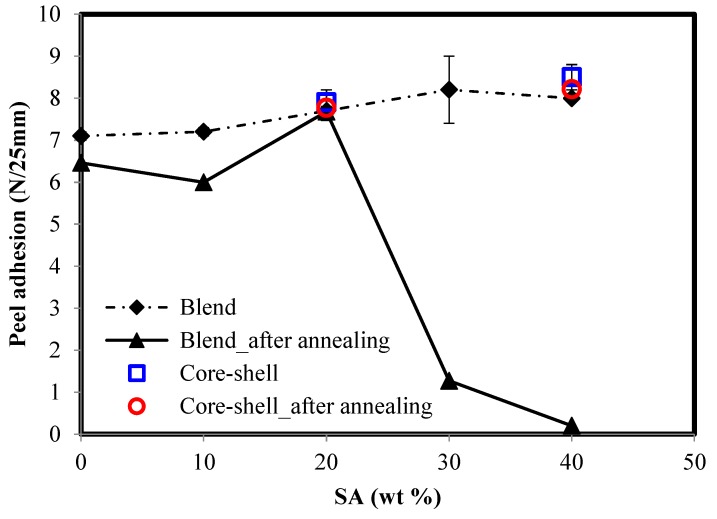
Effect of annealing of adhesive tapes cast from blends and core-shell latexes on their peel adhesion on stainless substrate.

First, the effect of the particle morphology on phase separation and migration and that of these phenomena on adhesive properties was checked on the adhesive tape without bonding to the stainless steel substrate (the adhesive tapes and the annealed adhesive tapes were used for probe tack and peel tests). Figure 1 shows that the work of adhesion of the blends with a poly(SA) content ≥ 30 wt % strongly decreased after annealing, whereas that of the core-shell was not affected by annealing. Similar behavior was found for the peel strength (Figure 2). Figure 2 also shows that the peel strength of the blends improves with the poly(SA) content and that annealing strongly reduced peel strength. There was a clear effect of annealing on the tackiness of the blend adhesive (Blend 5) is illustrated by Video S1 in Supporting Information. It has been reported that the crystallinity of polymer films containing poly(SA) domains was independent of annealing [23]. Therefore, the loss in adhesion after annealing cannot be the result of changes in crystallinity.

**Figure 3:**
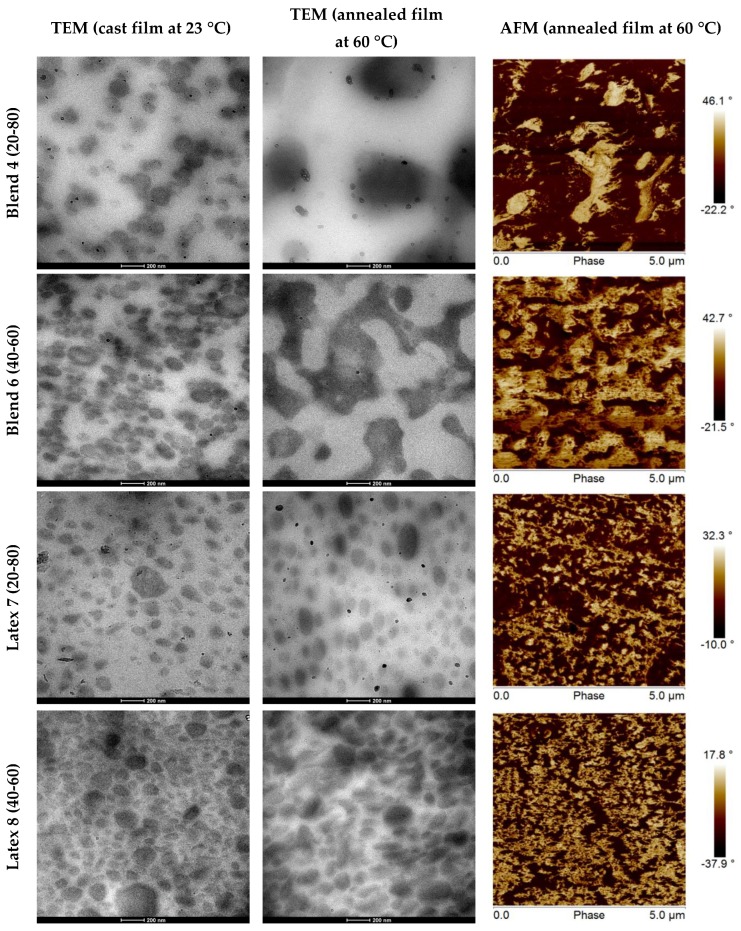
Cross-section TEM micrographs of the as-cast adhesive films at 23 °C (on the left side) and the TEM (in the middle) and AFM (on the right side) of the annealed adhesive films at 60 °C after cooling. The scale bar of TEM and AFM images are 200 nm and 5 µm, respectively.

The effect of annealing on film morphology is presented in Figure 3. This figure presents the TEM and phase AFM images of the cross-sections of the adhesive films cast from Blends 4 and 6 (SA/SC(M)A: 20/80 and 40/60) and core-shells of the same composition (Latexes 7 and 8). It is worth mentioning that the dark domains in TEM and bright domains in AFM micrographs are corresponding to poly(SA). Figure 3 clearly shows that a generalized phase migrations occurred for the blends whereas the films cast from core-shells did not show any significant migration. This confirms the hypothesis that blends facilitate phase migration.

**Figure 4:**
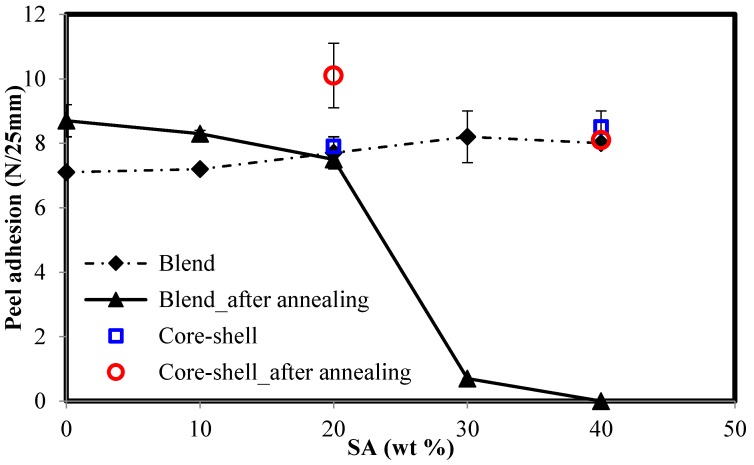
Effect of annealing of adhesives already bonded to stainless steel on peel adhesion.

**Figure 5:**
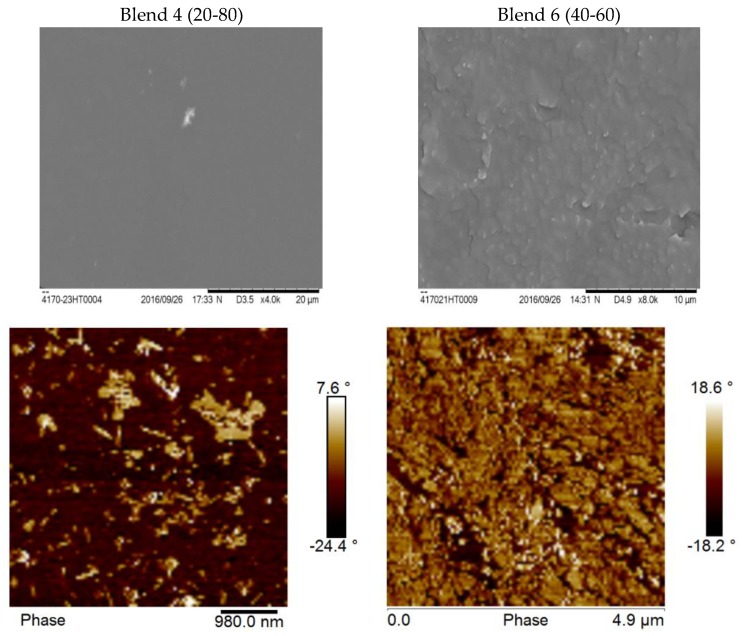
SEM and AFM phase images of SS-adhesive interface after detachment (the adhered tapes on SS annealed for 10 min at 60 °C and cooled to room temperature).

**Figure 6:**
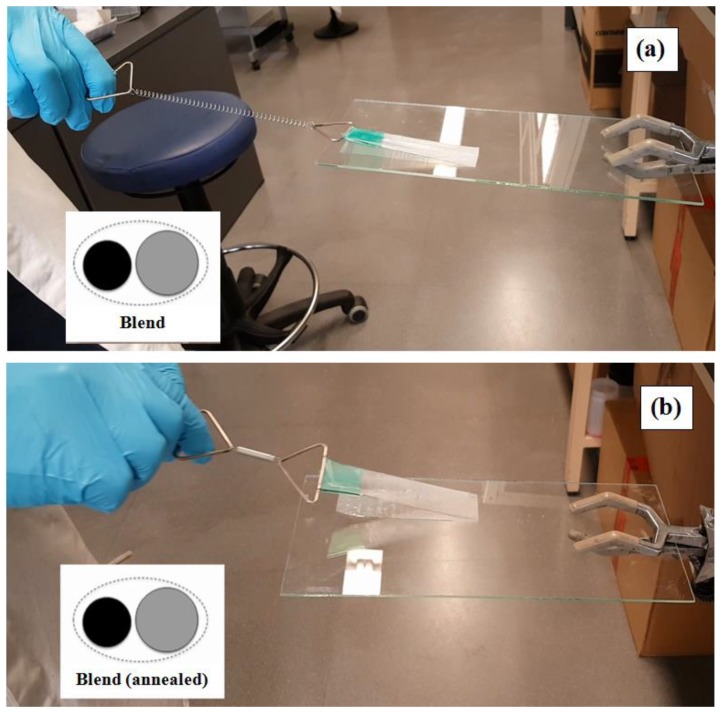
Effect of annealing on peeling for Blend 5. (**a**) Untreated bonded adhesive and (**b**) bonded adhesive annealed 10 min at 60 °C and cooled down.

The adhesive tapes were then applied on stainless-steel substrate and the peel strength was measured at 23 °C before and after annealing. The results are presented in Figure 4, where the behavior of the blends is compared with that of the core-shells. It can be seen that the peel strength of the blends containing ≥ 30 wt % of poly(SA) strongly decreased after annealing and cooling, whereas no effect was observed for the core-shells. The reason for this is the migration of the poly(SA) domains to the SS-adhesive interface, as observed in Figure 5. For concentrations of poly(SA) lower than 30 wt %, no effect of annealing was observed for the blends, likely because, as shown in Figure 5 there was not enough migration of the poly(SA) to the SS-adhesive interface. Moreover, the comparison of AFM cross-section phase image (Figure 3) and the AFM phase image of the substrate-film interface (Figure 5) of Blend 6 clearly show that the concentration of poly(SA) in the substrate-film interface is substantially higher than within the film which support this hypothesis that poly(SA) domains migrate toward substrate-adhesive interface. Video S2 in the Supporting Information and the pictures in Figure 6 illustrate the effect of annealing on peel strength for Blend 5 as the spring elongation was smaller after annealing. An important aspect that this figure shows is that the annealed adhesive presents an adhesive failure. This is due to the accumulation of the hard poly(SA) phase at the SS-adhesive interface.

Therefore, the waterborne pressure sensitive adhesives resulting from blending a hard semicrystalline polymer dispersion with a conventional (meth)acrylate PSA present the unusual and valuable characteristic of a high performance and an easy debonding with adhesive failure (i.e., without leaving residues on the substrate). It is worth mentioning that debondability of the adhesives can be modulated with a variety of crystallizable long side chain polymers [28] with melting temperature between −35 °C (poly(lauryl methacrylate)) to 60 °C (poly(behenyl acrylate)) and that the temperature range can be expanded (up to 135 °C) with side chain liquid crystalline polymers [29,30,31,32]. This opens the possibility to cover a broad range of applications.

## 4. Conclusions

In this work, a waterborne pressure-sensitive adhesive (PSA) that has the unusual and valuable combination of high adhesive performance and easy debondability on demand with adhesive failure is presented. The adhesive is prepared by blending a semicrystalline polymer dispersion with a conventional (meth)acrylate waterborne PSA. The semicrystalline particles provide cohesion improving the adhesive properties during use. On the other hand, when the PSA is annealed for a short period of time (10 min) at a temperature (60 °C) just above the melting temperature of the semicrystalline polymer (51 °C), massive migration of the melted polymer to the substrate-adhesive interface occurs. Upon cooling to room temperature, the melted polymer crystallizes becoming non-tacky and debonding occurs. It has been demonstrated that migration is a necessary condition and that particle morphologies that hinder the migration of the mobile polymer (e.g., core-shell morphologies with the semicrystalline polymer in the core) do not show debondability. The debonding temperature can be modulated between −35 °C and 135 °C by any different semicrystalline polymers.

## Figures and Tables

**Table 1:** Summary of latexes prepared.

Latex	SA/SC(M)A ^a^ (wt/wt)	Comment
1	100/0	batch miniemulsion
2	0/100	seeded semicountinuous emulsion copolymerization
3	10/90	Blends of latexes 1 and 2
4	20/80	
5	30/70	
6	40/60	
7	20/80	Core-shell latexes
8	40/60	

^a^ SC(M)A: 2EHA/MMA/MAA = 84/15/1 wt %.

## Supplementary Materials

The following are available online at http://www.mdpi.com/2073-4360/10/9/975/s1, Video S1: Effect of annealing on the tackiness of the blend adhesive (Blend 5), Video S2: Effect of annealing on peel strength for Blend 5.
